# Explaining symptoms in general practice: a predictive processing framework for primary care consultations

**DOI:** 10.1080/02813432.2026.2731974

**Published:** 2026-09-19

**Authors:** Kees van Boven, Peter Lucassen, Tim Olde Hartman, Omer Van den Bergh

**Affiliations:** a Department of Primary and Community Care Radboudumc, Nijmegen, Gelderland, The Netherlands; b Health Psychology, University of Leuven, KU Leuven, Belgium

**Keywords:** Predictive processing, symptom explanation, primary care, general practice, biopsychosocial model, consultation, persistent physical symptoms

## Abstract

**Aims:**

To present predictive processing as a clinically useful framework for explaining symptom variability in general practice consultations.

**Methods:**

Development of a conceptual framework through an iterative process of theoretical synthesis and clinical translation, informed by a supporting literature search integrating literature on predictive processing and symptom perception with primary care literature on consultation practice and persistent physical symptoms.

**Results:**

Predictive processing conceptualises symptom perception as an interaction between bodily signals and the brain’s prior expectations. Symptoms remain real and potentially disabling even when pathology is stable or minimal. The framework provides a structured approach for consultations: validating patient experiences, explaining symptom persistence, and guiding recovery-oriented management. A worked consultation example illustrates clinical application.

**Conclusions:**

Predictive processing offers a coherent framework to help general practitioners explain symptoms in primary care. Its limitations, including restricted prescriptive guidance and limited direct trial evidence, are acknowledged. The framework supports patient understanding and guides management focused on gradual recovery.

## KEY MESSAGES

- Patients with persistent physical symptoms in the absence of clear pathology frequently feel misunderstood; general practitioners lack a practical explanatory model that both validates and explains.
- Predictive processing offers a generalisable mechanism through which symptom experience can be explained without medicalising or psychologising.
- Clear explanation in the consultation reduces patient uncertainty, supports adaptive coping, and guides graduated recovery without unnecessary investigation.

## WHAT THIS PAPER ADDS

- Integrates predictive processing theory with the biopsychosocial approach, specifically applied to consultation practice in general practice.
- Provides a worked consultation example (ankle pain) illustrating practical application of the framework.
- Identifies the limitations and evidence gaps of predictive processing as a clinical framework, and outlines priorities for future research in primary care.

## Background

Symptoms are the primary reason patients consult general practitioners, and they form the central substance of primary care work [1]. In many consultations, thorough clinical assessment and appropriate investigation do not reveal serious or progressive disease [1,2]. Nevertheless, symptoms may remain severe, persistent, or fluctuating, significantly limiting daily functioning [3,4]. This mismatch between symptom burden and measurable pathology is one of the most common challenges in general practice, a challenge not fully addressed by existing clinical frameworks [3,4].

From the patient’s perspective, persistent physical symptoms without a clear diagnosis frequently generate a sense of not being believed or understood. Patients describe frustration when their experiences are dismissed as ‘nothing serious’ or implicitly attributed to psychological causes [5]. The absence of a satisfying explanation may itself perpetuate distress, drive repeated consultations, and lead patients to seek reassurance through further investigation [6]. Qualitative research consistently shows that patients with persistent physical symptoms report feeling that their suffering is not fully acknowledged, a perception that damages the therapeutic relationship and impedes recovery [5,7].

From the general practitioner’s perspective, the challenge is equally real. Once serious disease has been excluded, GPs are often left without a coherent explanatory model to offer [8]. Reassurance based solely on normal findings, ‘your tests are fine’, frequently fails to satisfy patients and may even be counterproductive if it leaves the cause of symptoms unexplained [9,10]. Telling a patient that their symptoms are ‘stress-related’ or ‘functional’ is often perceived as dismissive and may be experienced as stigmatising. GPs need an explanatory framework that is scientifically grounded, practically usable in brief consultations, and capable of validating symptom experience while remaining consistent with biomedical assessment.

The biopsychosocial model recognises that biological, psychological, and social factors influence health [11], but does not specify how these interact at the level of moment-to-moment symptom perception. Psychological explanations, when offered, are frequently communicated ineffectively and may fail to address patient concerns or validate their experience [5]. Pain neuroscience education (PNE) has demonstrated benefit for musculoskeletal pain, but its application to other symptom domains remains limited [12]. Cognitive-behavioural therapy (CBT)-based interventions have demonstrated effectiveness for persistent physical symptoms, but typically require specialist delivery and are difficult to integrate into brief general practice consultations, leaving GPs without a practical explanatory framework for use within the consultation itself. Lighter, GP-deliverable CBT-informed communication tools, such as one tested in a Norwegian cluster randomised trial, show that simplified versions can nonetheless be feasible within routine consultations [13,14]; the framework proposed here offers a complementary explanatory mechanism rather than a structured communication protocol. A related Oslo-based synthesis further outlines evidence-informed pathways for validating and explaining persistent physical symptoms in primary care, broadly consistent with the approach developed here [15]. In primary care systems with a strong gatekeeping and continuity function, including Scandinavian countries, the Netherlands, and the United Kingdom, general practitioners function as both first-contact clinicians and long-term coordinators of care. This dual role creates pressure to provide meaningful, non-alarming explanations, and to avoid unnecessary onward referral and investigation, concerns central to ongoing debates about overdiagnosis and healthcare sustainability [3,4].

Predictive processing (PP) addresses this gap by offering a mechanistic account of how physiological signals, prior illness experiences, and contextual factors interact to shape what patients feel [16–18]. Originating in computational neuroscience [16,17], the framework has been applied to pain, persistent physical symptoms, and the interpretation of internal bodily signals [16,18], but its application in consultations in general practice remains limited.

## Aims

The aim of this paper is to present predictive processing as a clinically useful conceptual framework for explaining symptoms in general practice consultations. Specifically, we seek to: (1) describe the core mechanisms of predictive processing as they apply to symptom perception; (2) translate these mechanisms into practical consultation principles, illustrated by two clinical examples; and (3) situate the framework in relation to existing approaches, identifying both its contributions and its limitations. The emphasis throughout is on consultation practice, patient understanding, and the reduction of unnecessary diagnostic investigation.

## Methods

This paper presents a conceptual framework for symptom explanation in general practice, developed through an iterative process of theoretical synthesis and clinical translation. The aim was not to conduct a systematic review of the literature but to develop a practically applicable framework grounded in established theory and relevant empirical evidence. The methodology is therefore best characterised as conceptual framework development, with a supporting literature search to inform and substantiate the framework’s components.

To identify relevant theoretical and empirical foundations, we searched PubMed, PsycINFO, and Google Scholar using search terms including ‘predictive processing’, ‘active inference’, ‘interoceptive inference’, ‘symptom perception’, ‘illness cognition’, ‘persistent physical symptoms’, ‘medically unexplained symptoms’, ‘primary care consultation’, and ‘pain neuroscience education’, in various combinations. We searched without language restriction and without a formal date limit, prioritising recent literature while including foundational theoretical texts. We focused on reviews, theoretical papers, and overviews rather than individual primary studies, on the grounds that these best capture the state of established knowledge in each domain. No formal inclusion or exclusion criteria were applied; selection was guided by relevance to the two core domains: (1) the theoretical and empirical literature on predictive processing and symptom perception [16–18] ,including interoceptive inference, the brain’s predictive interpretation of internal bodily signals, and symptom amplification, the tendency to attend to and interpret bodily sensations as more intense or threatening than warranted [19]; and (2) the primary care literature on consultation management of persistent physical symptoms, patient communication, and the effectiveness of existing explanatory approaches [8,11, 12,20,21].

The framework was developed iteratively: theoretical mechanisms were translated into consultation principles, which were then refined against the practical demands of brief primary care consultations. The selection of literature was not exhaustive and inevitably reflects the authors’ interpretive judgements. The resulting framework is illustrated with two consultation examples (Box 3 and Box 4) and supported by two clinical reference tools (Box 1 and Box 2). It is presented as a basis for clinical consideration and future empirical evaluation, not as a synthesis of a defined body of evidence.

## Results

### The predictive processing model of symptom perception

Predictive processing treats symptoms as perceptual constructions: active inferences made by the brain about the state of the body [16]. The brain does not passively receive signals from the body but continuously generates predictions about likely bodily states, based on prior experience and contextual cues. Incoming sensory signals are compared against these predictions. When signals are not discrepant from predictions, perception largely overlaps with those predictions. Where signals violate predictions, generating ‘prediction errors’, the brain updates its model of the body’s state. Symptoms are the product of this process: not direct readouts of tissue pathology, but the brain’s best estimate of bodily state given the available evidence.

That evidence includes peripheral signals from nociceptors and interoceptors, but also prior illness experiences, recovery expectations, and the broader context in which symptoms occur, including the responses of family members and significant others [16,17]. A key concept is the role of prior beliefs, or ‘priors’: the brain’s accumulated expectations about the body, shaped not only by individual experience but also by cultural beliefs and lay narratives about illness, that shape perception automatically and mostly outside awareness. A patient who has previously experienced a myocardial infarction may develop a strong prior associating chest discomfort with life-threatening danger. The same physiological signal that another person experiences as minor tension may, in this patient, be perceived as intense and alarming, not because the peripheral signal differs, but because the brain’s interpretive framework does. When a prior holds that a sensation is dangerous, that recovery is unlikely, or that activity will worsen symptoms, it actively shapes what is perceived.

#### A further concept is ‘precision weighting’

The brain continuously judges how much trust to place in incoming bodily signals versus its own prior expectations. This process of weighing internal bodily signals against expectation is what is meant by interoceptive inference. In states of heightened anxiety or uncertainty, this balance shifts so that the brain relies more heavily on its expectations and becomes more sensitive to ambiguous signals, making them more likely to be interpreted as significant and threatening. The effect is most pronounced when peripheral signals are weak, ambiguous, or fluctuating, as often occurs in post-infectious states and functional syndromes, the brain relies more heavily on priors, and symptom intensity may be driven more by expectation than by detectable peripheral change. This pattern is recognisable across presentations common in primary care: patients with post-COVID fatigue whose exhaustion persists beyond resolution of the acute infection [20], patients with post-infectious syndromes more broadly [22], patients with functional dyspepsia whose symptom severity fluctuates independently of endoscopic findings [23], and patients with chronic low back pain whose disability correlates poorly with radiological findings [24]. These examples share a common mechanism: persistent or non-specific input from the body, whether residual physiological change, stress responses, or poorly understood sensations, interacts with the brain’s predictive model and strong prior expectations to shape what is perceived. Neither peripheral signals nor priors alone determine the outcome; it is their interaction, weighted by the brain’s assessment of reliability, that drives symptom experience. And it is precisely this interaction that the consultation can address.

### Implications for general practice consultations

*Translating predictive processing into consultation language* is one way the framework can be applied in practice. The framework provides a set of principles that can be integrated naturally into routine consultations, once serious disease has been excluded and safety-netting is in place: effective explanation, gradual activity, and avoidance of unnecessary testing [25].

Most immediately, explanation itself becomes part of treatment. Rather than concluding with ‘nothing was found,’ the clinician can explain that reassuring results indicate nothing dangerous or progressive, while acknowledging that symptoms reflect the body’s current physiological state and the brain’s ongoing interpretation of it. This reframing reduces uncertainty, which is itself a driver of symptom amplification. Patients who develop a coherent, non-threatening account of their symptoms are better positioned to engage with management and less likely to seek repeated reassurance through further investigation [6,25]. It is important to verify with the patient whether the explanation has in fact reduced their uncertainty, and to adjust accordingly.

#### Gradual activity warrants careful framing

Paced activity here means a graded, symptom-informed increase in activity level, rather than either complete rest or pushing through symptoms. In predictive processing terms, cautious resumption of activity provides the brain with new evidence: that movement is safe, that tolerance is possible, that priors about danger require updating. This is distinct from simply ‘pushing through’ symptoms. Explaining this distinction to patients, that paced activity actively recalibrates the predictive model, can improve engagement with graduated management and reduce fear-avoidance behaviour [12].

#### A third consideration is the avoidance of unnecessary investigation

Repeated testing in the absence of new clinical indicators can inadvertently reinforce illness priors, signalling to patient and brain alike that something remains wrong. Where safety-netting is clear and findings are reassuring, the predictive processing framework supports a measured approach to further investigation, reducing the risk of entrenchment of symptom-maintaining beliefs [25].

Box 1. Key consultation principles: what predictive processing adds to primary care reasoning

- Validate the symptom experience. Frame symptoms as real bodily experiences, originating from brain processes, not as indicators of tissue damage or a sign of personal failing.
- Link normal findings to safety. Reassuring results mean nothing dangerous or progressive, not that symptoms are imagined or trivial.
- Explain physiological persistence. Symptoms can continue during recovery as the brain’s predictive model recalibrates; this is expected and does not indicate worsening.
- Frame graded activity as therapeutic. Paced activity provides the brain with new information about safety and tolerance; it updates the prior.
- Limit investigation to clinical need, and safety-net clearly. Unnecessary testing reinforces illness priors; where findings are reassuring, specify which changes warrant reassessment so the patient can distinguish normal recovery from genuine warning signs.

Box 2. Example consultation language: a predictive processing perspective

*On normal findings:*

- “Your tests do not show anything dangerous, which is reassuring. It does not mean your symptoms are not real—they are. It means your body is working to settle, and your brain is still interpreting signals cautiously.”

*On persistence:*

- “It is common for symptoms to continue for a while even when nothing serious is wrong. The brain takes time to update its picture of the body, that is a normal part of recovery, not a sign that something has been missed.”

*On gradual activity*:

- “The idea is not to push through the symptoms, but to give your brain new information that movement is safe. Small, consistent steps do that better than rest alone.”

*On safety-netting*:

- “If you notice [specific symptom changes], come back and we will reassess. Otherwise, what you are experiencing fits with what we would expect at this stage.”

### A worked example: persistent ankle pain

The following case illustrates how predictive processing can be applied in a general practice consultation with a patient presenting with persistent pain in the absence of identifiable ongoing pathology.

Box 3. Consultation example: unexplained ankle pain

Case

A 38-year-old woman presents with persistent pain and stiffness in her left ankle, nine months after an inversion injury. Plain radiographs and ultrasound showed no structural damage. She has completed a full course of physiotherapy; the pain has improved somewhat but continues to limit her daily functioning. She is concerned that something has been missed and feels that nobody believes her.

*Explanation*

Following validation of the patient’s symptoms and discussion of the investigation findings, the general practitioner might say the following:

- “The scans and ultrasound show that the tissue has healed well, there is no longer any damage that explains your pain. That is a reassuring result. But I understand that it is confusing, because the pain is still there, and it is real.”
- “What is most likely happening is this. Your ankle sent pain signals for a long time, rightly so, because there was an injury. Your brain learned from that: it built up a strong expectation that the ankle is in danger and needs to be protected. Now that the tissue has healed, the ankle itself is sending fewer alarm signals. But the brain does not automatically update its expectation. It keeps interpreting cautiously, and that is why you continue to feel pain, even though nothing is broken any more.”
- “You can help the brain revise that expectation. Not by ignoring the pain, but by gradually showing it that movement is safe. Each time you do something and find that it is manageable, you are giving your brain new information. That is exactly what the next phase of rehabilitation is designed to achieve.”
- “If you do notice new symptoms, swelling, redness, or a different kind of pain from what you have been experiencing, I would like you to come back. But what you are feeling now fits with this recovery process.”

*Commentary*

This explanation validates the symptom, links normal findings to safety, and frames rehabilitation as evidence that updates the brain’s predictive model, rather than an effort to override pain.

Box 4. Extending the framework beyond pain: persistent fatigue

Case

A 44-year-old man presents with persistent fatigue six months after a confirmed viral infection that has otherwise fully resolved. Blood tests and further work-up are unremarkable. He struggles to link his fatigue to any ongoing physical cause and fears his GP will attribute it to ‘being stressed’.

*Explanation*

- “Your tests are reassuring, there is no ongoing infection or other disease process driving this. What is more likely is that your body, quite reasonably, learned to protect itself while you were acutely unwell, and your brain is still generating fatigue signals as a precaution, even though the original threat has passed.”
- “This is not a sign that something has been missed, and it is not ‘just in your head’—it is a normal, if unhelpful, side effect of how the brain protects the body. You can help it update that expectation through paced activity: a graded, symptom-informed increase in what you do, rather than resting completely or pushing through until you crash.”

*Commentary*

This example illustrates that the same predictive mechanism described for pain applies to fatigue, where there is no visible ongoing injury to point to—and shows paced activity applied outside a musculoskeletal context.

## Discussion

### Situating predictive processing within existing frameworks

Predictive processing complements and extends the explanatory models currently available in primary care (Table 1). The biopsychosocial model identifies the relevant domains, biological, psychological, and social, but does not specify how these interact at the level of symptom perception [11]. Pain neuroscience education targets pain-related beliefs effectively, but its application has been largely confined to musculoskeletal presentations [12]. Cognitive-behavioural models of persistent physical symptoms address illness cognitions and behaviours, and CBT-based interventions have demonstrated significant improvements in primary care patients [21]; however, these approaches typically require specialist delivery and are difficult to integrate into brief general practice consultations. Lighter, GP-deliverable CBT-informed tools are an exception, as noted above [13]. Predictive processing offers a single, generalisable mechanism that operates across symptom types and conditions, without requiring a different explanatory framework for each diagnostic category.

**Table 1.** Predictive processing compared with existing explanatory models in general practice.

Framework	Strengths	What predictive processing adds
Biopsychosocial model	Broad and inclusive; acknowledges multiple domains	Does not specify how biological, psychological, and social factors interact at the level of symptom perception. PP specifies this mechanism directly.
Pain neuroscience education	Effective for pain-related beliefs; well evidenced	Largely confined to musculoskeletal pain; not readily generalisable to other symptom domains. PP generalises the same mechanism to other symptom domains.
CBT/illness perception models	Targets cognitions and behaviour; demonstrated effectiveness	Typically requires specialist delivery; difficult to integrate into brief general practice consultations. PP offers a brief, GP-deliverable mechanism usable within the consultation itself.
Predictive processing	Generalisable mechanism; non-stigmatising language; applicable within the consultation	Primarily descriptive; limited direct RCT evidence for PP-based explanation in clinical consultations

### *Relevance for primary care systems* with a gatekeeping and continuity function

Primary care systems with strong gatekeeping and continuity, including Scandinavian countries, the Netherlands, and the United Kingdom, are particularly relevant for the predictive processing framework because general practitioners often maintain long-term therapeutic relationships with patients. This continuity gives GPs a unique position to identify and reshape illness priors, but it may also reinforce patients’ expectations that symptoms reflect unresolved disease when explanations are inconsistent, or investigations are repeated without clear added value.

The framework is compatible with the emphasis on patient participation and shared decision-making that characterises these healthcare cultures, positioning the patient as an informed participant in recovery rather than a passive recipient of investigation or reassurance.

The gatekeeping role of GPs in these systems means that decisions about investigation, referral, and reassurance carry significant downstream consequences for healthcare utilisation. The predictive processing framework aligns with ongoing European and international policy concerns about overdiagnosis and medicalisation of common symptoms [3,4]. By providing a credible, mechanistically grounded explanation for symptom persistence, GPs can more confidently avoid investigative cycles that are unlikely to benefit the patient and can do so in language that is transparent and respectful rather than dismissive.

## Limitations and future directions

 

- As a conceptual framework, predictive processing is descriptive rather than prescriptive and should inform, not replace, clinical judgement about when to rely on graded activity, cognitive interventions, pharmacological treatment, or specialist referral. Several further limitations warrant attention.
- Individual variation in predictive processing cannot be quantified in routine primary care. The relative contribution of prior beliefs versus peripheral signals, or of ‘top-down’ prediction versus ‘bottom-up’ error, is not directly measurable with current instruments, though several questionnaires can provide relevant indications [19,26,27]. Concepts such as precision weighting remain background theory rather than clinical parameters. The framework also presupposes careful exclusion of serious disease but cannot eliminate the residual risk of missed diagnosis: once a predictive processing explanation has been adopted, new or evolving symptoms might receive less attention. Robust safety-netting and periodic review therefore remain essential, and the framework should be understood as an adjunct to, not a substitute for, thorough diagnostic reasoning.
- The therapeutic mechanisms and empirical effects of PP-informed explanations in primary care are not yet well established.Structured, PP-informed consultation strategies have not themselves been evaluated in primary care. It remains uncertain how individual patients respond to brain-based symptom explanations; some may still experience these as psychologising, despite the non-stigmatising framing. Existing work supports the broader role of expectations, illness perceptions, and prior experiences in shaping symptom outcomes [18,26,28], and related interventions have shown benefit [20,21], but structured PP-based explanations in real-world general practice consultations have not been rigorously evaluated. The closest indirect evidence comes from GP-delivered structured communication tools such as ICIT, which have shown benefit for related outcomes without being framed in predictive-processing terms [13]; It remains unclear to what extent any benefit derives from the specific theoretical account versus more generic factors such as validation and reduction of uncertainty, a question directly raised by the overlap between PP-based explanation and placebo/nocebo mechanisms [29]. Priority questions for future research include how predictive processing can be taught effectively to GPs, how acceptable brain-based explanations are to different patient groups, whether PP-informed communication improves understanding, reassurance, or healthcare utilisation compared with usual care, and what training this requires across undergraduate, postgraduate, and continuing GP education. The GP’s own communication style and the continuous therapeutic relationship may further modulate the effectiveness of PP-informed explanations, consistent with broader evidence on provider characteristics and expectancy effects [30].
- The selection of literature was not exhaustive and inevitably reflects the authors’ interpretive judgements. The consultation language in Box 2 and Box 3 has not been empirically validated; the framework is presented for clinical consideration and as a basis for future empirical work, not as established evidence-based guidance.

## Conclusions

Primary care is fundamentally concerned with symptoms rather than established disease, and the relationship between symptoms and pathology is rarely proportional [1,3,4]. Patients with persistent physical symptoms without clear pathological explanation frequently feel unheard; general practitioners often lack a coherent framework with which to respond. Predictive processing offers a mechanistic account of why symptoms persist: shaped not only by peripheral signals but by the brain’s interpretive framework, which is itself influenced by prior illness experience, recovery expectations, and what is communicated in the consultation.

The framework identifies specific points of clinical leverage: the language used to interpret test results, the framing of gradual activity, and the deliberate avoidance of investigative cycles that may entrench rather than resolve symptoms. Together, these move the consultation from a process of exclusion towards one of active explanation and graduated management. Predictive processing as a theoretical account has already undergone extensive empirical testing; what has not yet been tested is its use as a communication strategy in primary care consultations, which we present here as a promising avenue that now warrants empirical evaluation.

For general practitioners working in primary care systems with a strong gatekeeping and continuity function, where the sustained management of undifferentiated presentations is central, predictive processing offers a valuable, scientifically grounded, and patient-centred addition to the explanatory toolkit.

## Data Availability

No dataset was generated or analysed during the development of this conceptual framework. Data sharing is not applicable to this article.
